# The Charge Storage Mechanism and Durable Operation in Olivine–Lithium–Iron–Phosphate for Mn‐based Hybrid Batteries

**DOI:** 10.1002/advs.202502866

**Published:** 2025-03-17

**Authors:** Jangwook Pyun, Hyungjin Lee, Hyeonjun Lee, Sangki Lee, Seunghyeop Baek, Hyeju Kwon, Seung‐Tae Hong, Munseok S. Chae

**Affiliations:** ^1^ Department of Nanotechnology Engineering Pukyong National University Busan 48547 Republic of Korea; ^2^ Department of Energy Science and Engineering DGIST Daegu 42988 Republic of Korea; ^3^ Department of Chemistry and Chemical Biology University of New Mexico New Mexico 87131 USA

**Keywords:** aqueous battery, aqueous electrolyte, hybrid ion battery, LiFePO_4_, manganese hybrid batteries

## Abstract

Aqueous batteries have garnered considerable attention because of their cost‐effectiveness, sufficient capacity, and non‐flammable water‐based electrolytes. Among these, manganese batteries are particularly attractive owing to their stability, abundance, affordability, and higher energy density. With a lower redox potential (Mn: −1.19 V vs SHE) than zinc (Zn: −0.76 V vs SHE), manganese batteries theoretically offer superior energy density over traditional zinc‐based systems. In this study, LiFePO_4_ is introduced as a cathode material in aqueous manganese‐based hybrid batteries for the first time. Through electrochemical characterization and advanced structural and spectroscopic analyses, the charge storage mechanisms of protons in to the FePO_4_ are elucidated. Cation diffusion pathways are also investigated via diffusion barrier calculations. This study presents manganese hybrid batteries with a good stability and capacity of ≈109.2 mAh g^−1^ at 40 mA g^−1^, alongside a cycle retention of 42.1% after 3000 cycles at 320 mA g^−1^. Furthermore, an Mn^2+^/Li^+^ hybrid battery, achieving ≈1.6 V and superior durability (81.5% @ 1000th), is proposed.

## Graphical abstract

The study presents LiFePO_4_ as a cathode material for Mn^2+^/H^+^ hybrid batteries, detailing its charge storage mechanism in Mn^2^⁺ aqueous electrolytes. Furthermore, an Mn^2^⁺/Li⁺ hybrid battery system is introduced, demonstrating its excellent electrochemical performance and durability.

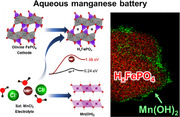

## 1 Introduction

Rechargeable batteries using aqueous electrolytes are emerging as a promising alternative to Li^+^ batteries, particularly because of their enhanced stability, cost‐effectiveness, and environmental benefits.^[^ ^1^ ^]^ The use of water‐based electrolytes substantially lowers the risks of fire and explosion, making them highly suitable for a wide range of large‐scale energy storage applications. The intrinsic safety and minimal environmental footprint of these batteries align with the growing global emphasis on sustainable and green energy technologies. Historically, aqueous rechargeable batteries have used metal anodes such as Fe,^[^ ^2^ ^]^ Zn,^[^ ^3^ ^]^ and Al^[^ ^4^ ^]^ because of their inherent stability and high theoretical specific capacities. However, these anode materials exhibit certain drawbacks, such as the low operating voltage of Fe and the elevated polarization resulting from the formation of a passivation layer on Al.^[^ ^5^ ^]^ Even Zn‐based secondary batteries, considered the most advanced among aqueous systems, face limitations in reaching high energy densities due to the relatively high redox potential of Zn (−0.76 V vs SHE).^[^ ^6^ ^]^

Aqueous Mn‐based batteries present a promising alternative, offering several advantages, including high theoretical gravimetric and volumetric capacities (976 mAh g^−1^ and 7250 mAh cm^−^^3^), abundance in the Earth's crust, cost‐effectiveness, and a low redox potential (−1.19 V vs SHE).^[^ ^7^ ^]^ The low redox potential of Mn enables higher battery operating voltage compared with Zn‐based batteries. Moreover, they can be safely assembled in ambient conditions, enhancing process efficiency. Despite these benefits, the development of Mn‐based storage host structures has been relatively underexplored, primarily due to the divalent charge of Mn ions. Some progress has been made; however, few electrode materials have been explored for reversible operation in Mn‐based electrolytes,^[^ ^7d^ ^]^ including Mo_6_S_8_,^[^ ^8^ ^]^ V_2_O_5_,^[^ ^8^ ^]^ Ag_0.33_V_2_O_5_,^[^ ^9^ ^]^ NiHCF,^[^ ^8^ ^]^ Al_0.1_V_2_O_5_·1.5H_2_O,^[^ ^10^ ^]^ Mn_0.18_V_2_O_5_·nH_2_O,^[^ ^11^ ^]^ and organic electrodes such as PTCDA and coronene.^[^ ^7d^ ^]^ Developing high‐voltage Mn‐based batteries remains challenging, as most existing cathode materials operate at voltages below 1 V (vs Mn/Mn^2^⁺). Prussian blue structures typically exhibit low charge/discharge capacities of less than 100 mAh g^−1^, while vanadium‐based materials primarily operate within the 0.8–1.1 V (vs Mn/Mn^2^⁺) range due to their low redox potential. This highlights the urgent need for novel cathode materials specifically designed for Mn‐based batteries to achieve improved performance at higher voltages. In particular, iron‐based materials, with their higher redox potential compared to vanadium, are considered promising candidates for this purpose. Nevertheless, iron‐based cathode materials have not yet been reported as cathodes for manganese‐based batteries.

**Figure 1:**
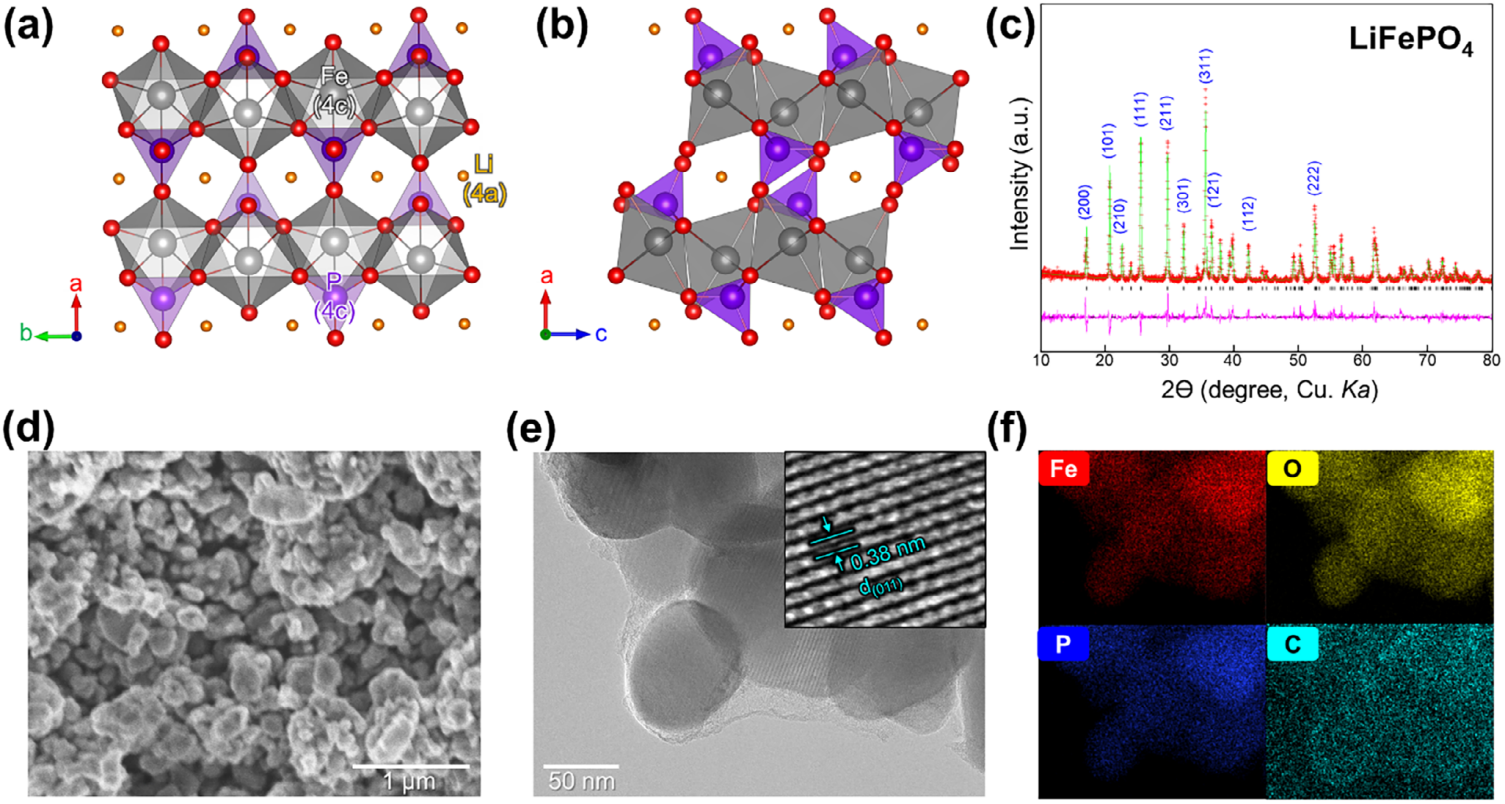
Characterization of LiFePO_4_ cathode: Crystal structure of LiFePO_4_ along the a) ab‐plane, b) ac‐plane. c) Rietveld refinement results from XRD. d) SEM image. e) TEM image and high‐resolution lattice fringe (inset). f) TEM‐EDX elemental mapping for Fe (red), O (yellow), P (blue), and C (cyan).

Olivine‐structured LiFePO_4_ (LFP) (**Figure** **1**a,b) is considered one of the most promising materials for low‐cost battery systems owing to its abundant availability, affordability, and exceptional thermal, chemical, and electrochemical stability. LFP has been widely studied for various metal cations (Li⁺,^[^ ^12^ ^]^ Na⁺,^[^ ^13^ ^]^ Mg^2^⁺,^[^ ^14^ ^]^ and Ca^2^⁺^[^ ^15^ ^]^) owing to its open framework, which can accommodate cations with a broad range of ionic radii. Therefore, LiFePO₄, which is widely used as a cathode material for lithium‐ion batteries, presents a promising option, as it can be produced cost‐effectively with well‐established manufacturing processes. This material typically exhibits an operating voltage of ≈3.4 V (vs Li/Li⁺), making it suitable for high‐voltage aqueous systems. Consequently, several studies have focused on Zn/LFP‐based batteries, which demonstrate a working voltage of ≈1.2 V—insufficient for many applications.^[^ ^16^ ^]^ However, by incorporating Mn as the anode, which has a lower potential than Zn, developing a cost‐efficient, high‐voltage, and safe aqueous battery system becomes feasible. Nonetheless, no existing studies support the operation of olivine materials such as LFP in Mn‐based electrolytes, highlighting the need for further investigation. If an aqueous Mn battery is successfully developed, it could theoretically achieve an operating voltage of ≈1.6 V, offering a first step forward for practical energy storage systems.

This study presents, for the first time, the use of LFP as a cathode material in aqueous Mn^2+^/H^+^ hybrid batteries. Through electrochemical characterization, advanced structural spectroscopic analysis, and diffusion pathway calculations, we clarify the charge storage mechanisms involving mainly from protons. The LFP cathode demonstrates good stability, delivering a capacity of ≈109.2 mAh g^−1^ at 40 mA g^−1^, with a cycle retention of 42.1% after 3000 cycles at 320 mA g^−1^ in a saturated MnCl_2_ aqueous electrolyte. However, the cycling performance remains unsatisfactory. We propose a novel Mn^2^⁺/Li⁺ hybrid battery concept to address this limitation, achieving an operating voltage of ≈1.6 V and substantially improved durability, retaining 81.5% capacity after 1000 cycles. These results underscore the potential of Mn‐based batteries for future applications in aqueous energy storage systems.

## 2 Results and Discussion

### 2.1 Synthesis and Material Characterizations

Nanosized LFP was synthesized using a sol–gel method with LiOH, Fe(CH_3_COO)_2_, and NH_3_H_2_PO_4_. Detailed synthesis procedures are provided in the Supporting Information Figure S1 (Supporting Information). Powder XRD analysis confirmed the formation of an orthorhombic phase with a *Pmna* space group, exhibiting unit cell parameters of a = 10.327(1) Å, b = 6.006(1) Å, and c = 4.693(1) Å (Figure 1c). The Rietveld refinement results align with previous studies, with comprehensive crystallographic data provided in Table S1 (Supporting Information). The SEM image of the synthesized LFP powder (Figure 1d) reveals sub‐micron‐sized particles ranging from 50 to 200 nm in size (Figure S2, Supporting Information). High‐resolution TEM imaging (Figure 1e; Figure S3, Supporting Information, and inset) displayed clear lattice fringes corresponding to the d(011) spacing, ≈0.38 nm. We used energy‐dispersive X‐ray spectroscopy (EDX) via TEM elemental mapping for qualitative and quantitative elemental analysis. The mapping results confirmed the uniform distribution of Fe, O, P, and C throughout the synthesized powder (Figure 1f). SEM‐EDX analysis further corroborates the elemental composition of the pristine powder (Figure S4, Supporting Information). Based on various material characterization analyses, we confirm that our LFP was successfully synthesized without any impurities.

### 2.2 Electrochemical Charge Storage Performance

**Figure 2:**
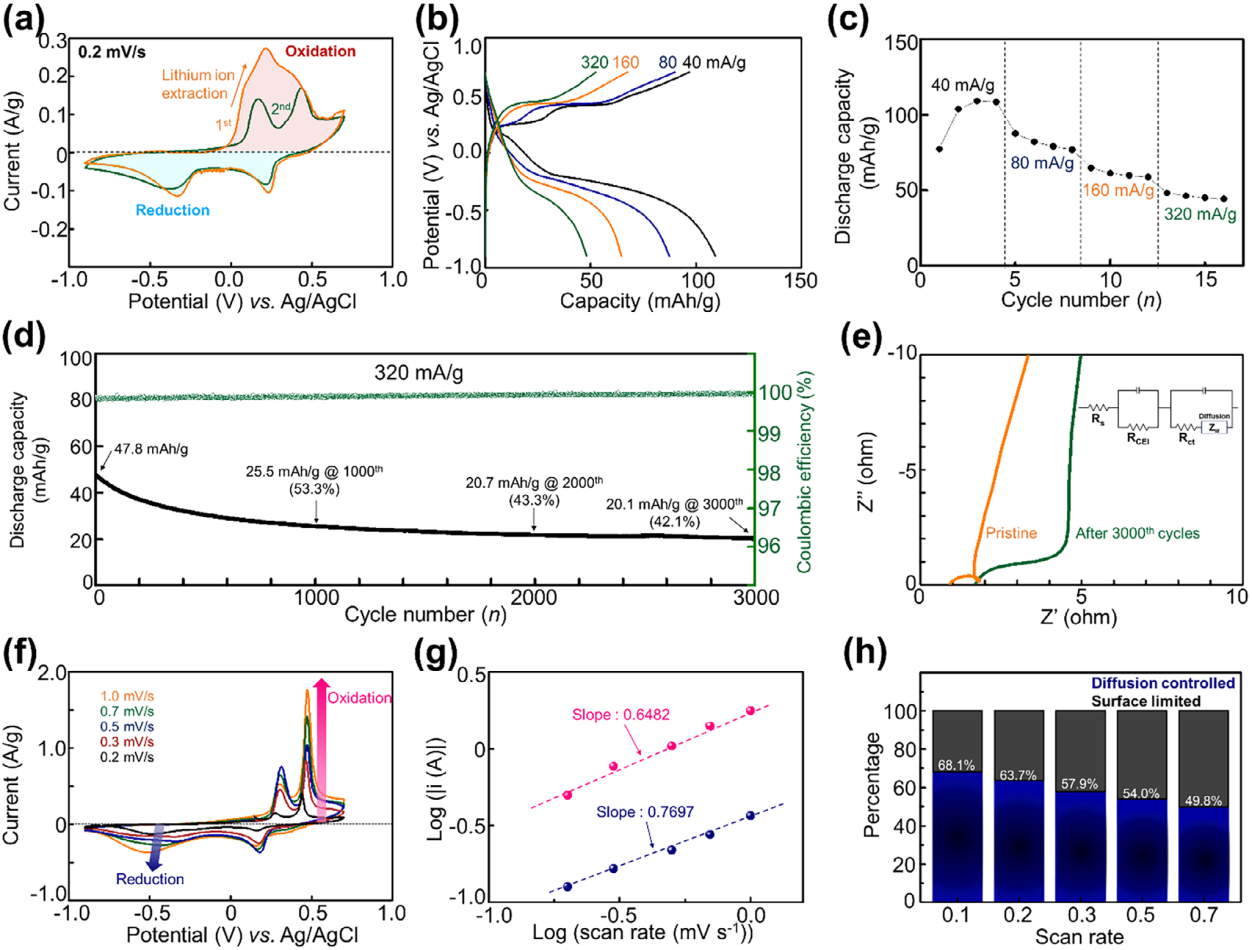
Electrochemical charge storage performance of LiFePO_4_: a) CV curve at a scan rate of 0.2 mV s^−1^. b) GCD curves at different current densities. c) Rate performance at various current densities. d) Long‐term cyclability at 320 mA g^−1^. e) Impedance spectra before and after 3000 cycles. f) CV curves at scan rates in the range of 0.2–1.0 mV s^−1^. g) Determination of *b*‐values from the relationship between the specific cathodic peak current and scan rate. h) Calculated ratio of diffusion‐controlled (orange) and surface‐limited (black) reactions for various scan rates.

All electrochemical tests were conducted using custom‐designed three‐electrode cells. The cyclic voltammetry (CV) curve at a scan rate of 0.2 mV s^−1^ revealed two primary redox peaks during the reversible cycles (**Figure** **2**a). During the initial charge process, Li ions were extracted from LFP. In subsequent cycles, Mn ions and protons became the dominant contributors to the reversible redox reactions. Two distinct discharge/charge peaks were observed at 0.22 and −0.36 V for discharge and at 0.17 and 0.43 V for charge (vs Ag/AgCl). Based on the reaction mechanisms detailed in the elemental analysis section, these complex peaks are attributed to a combination of proton intercalation, Mn surface adsorption, and Mn(OH)_2_ formation. The galvanostatic charge–discharge (GCD) profiles were recorded under various current densities (Figure 2b). A Li extraction process was applied before the system achieved reversible charge–discharge cycles (Figure S5, Supporting Information). The CV curves and GCD profiles exhibited similar behavior in terms of operating voltage and two‐step reactions. The discharge plaaou shows at the ≈0.20 and −0.30 V. As the current density increases, the redox process at 0.20 V becomes negligible, indicating that ion diffusion is faster in the −0.30 V region compared to the 0.20 V region. the LFP electrode demonstrated excellent rate capability, with reversible discharge capacities of 109, 87.4, 64.6, and 48.1 mAh g^−1^ at current densities of 40, 80, 160, and 320 mA g^−1^ under saturated MnCl_2_ electrolytes, respectively (Figure 2b). Figure 2c illustrates that the material maintained consistent electrochemical performance (capacity) throughout cycling. At a current density of 320 mA g^−1^, the discharge capacity after long‐term cycling began at 47.8 mAh g^−1^, retaining 42.1% of its capacity (20.1 mAh g^−1^) after 3000 cycles, demonstrating stable cycle retention (Figure S6, Supporting Information). Over 3000 cycles, the average Coulombic efficiency remained high at 99.96%.

Figure 2e presents the impedance spectra before and after 3000 cycles. The internal resistance of the cell (R_s_) increased slightly from 0.893 to 1.67 Ω, suggesting that side reactions during cycling minimally affected the interaction between the titanium current collector and the electrode materials and the stability of the Ti current collector and MnCl_2_‐based aqueous electrolyte. The charge‐transfer resistance (R_CEI_ + R_ct_) for the pristine electrode was measured at 0.949 Ω, rising modestly to 2.71 Ω after 3000 cycles, likely due to the accumulation of side products, such as Mn‐hydrated phases or Mn(OH)_2_, on the cathode surface. A slight increase in the Warburg slope indicated that diffusion kinetics became more active during the cycling of the cathode material.

CV tests were performed with various scan rates ranging from 0.2 to 1.0 mV s^−1^ to examine the cation intercalation behavior during the charge–discharge process. Figure 2f presents the results. The power‐law relationship was employed to analyze the cation storage mechanism, distinguishing between diffusion‐controlled and surface‐limited reactions.^[^ ^17^ ^]^ The power‐law exponent (b), which differentiates between capacitive and diffusion‐driven processes, was calculated to be 0.7697 and 0.6482 for the reduction and oxidation processes, respectively (Figure 2g; Figure S7, Supporting Information). These results indicate that diffusion and surface‐controlled mechanisms contribute to the overall reaction kinetics of LFP. Further analysis revealed that ≈68.1% of the cations intercalated into the host, whereas 32.9% exhibited surface‐adsorbed capacitive behavior, primarily attributed to the formation of an electrostatic double layer (Figure 2h). Moreover, as the scan rate increased, a significant rise in surface capacitance was observed (Figure 2h; Figure S7, Supporting Information), underscoring the growing contribution of surface‐driven phenomena at higher scan rates.

It was previously unknown whether the LFP electrode undergoes redox reactions in a manganese‐based electrolyte. However, through this study, we confirmed that LFP exhibits a two‐step redox reaction in saturated MnCl_2_ aqueous electrolyte. Furthermore, we found that the majority of charge storage occurs through an intercalation process. The electrode demonstrated stable operation over 3000 cycles, and impedance analysis before and after cycling revealed a slight increase in both internal resistance (IR) and charge transfer resistance.

### 2.3 Charge Storage Mechanism

**Figure 3:**
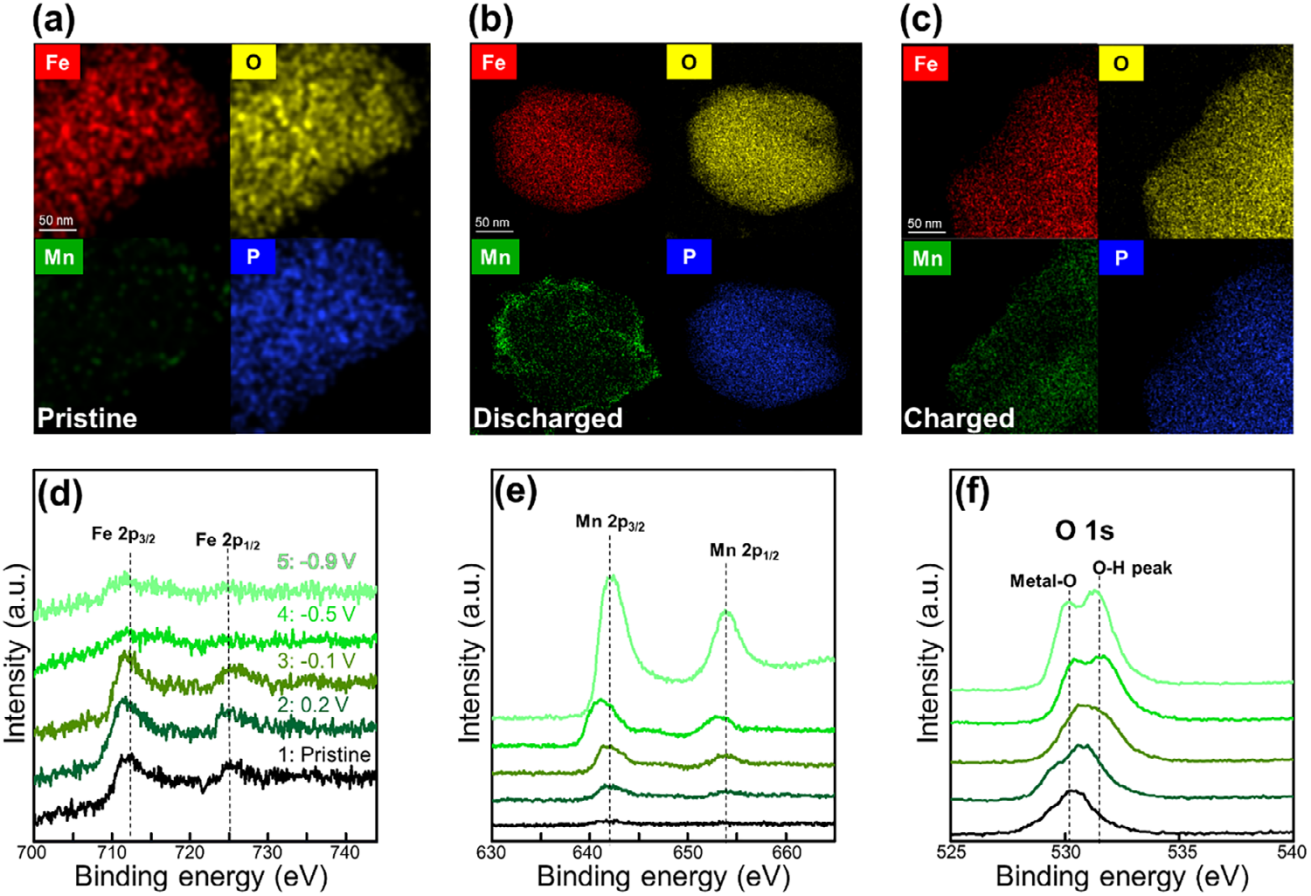
TEM‐EDX elemental mapping of a) pristine, b) fully discharged FePO_4_, and c) charged. XPS spectra of d) Fe 2p, e) Mn 2p, and f) O 1s at various discharge voltages.

Elemental mapping analysis during the discharge process was performed using TEM‐EDX (Themis Z) and XPS (X‐ray photoelectron spectroscopy, ESCALAB 250Xi). **Figure** **3**a–c illustrates the electrode in its pristine (LFP), fully discharged (−0.9 V vs Ag/AgCl), and recharged (0.7 V vs Ag/AgCl) states, respectively. In the pristine state, Mn was absent but was uniformly distributed upon full discharge, indicating that the redox reaction may involve the Mn ions (Figure S8, Supporting Information). However, residual Mn was detected after recharging, suggesting the possibility of an irreversible reaction from the Mn electrolyte and generation of the Mn(OH)_2_ formation (Figure S9, Supporting Information). The Mn signal was particularly strong on the electrode surface after discharge (Figure S10, Supporting Information), which could be attributed to proton intercalation and the formation of Mn(OH)₂ on the LFP surface.^[^ ^9^ ^]^

The proposed reaction mechanism is outlined below.

Step 1.

(1) $$Mn^{2+}\;\left(\mathrm{aq}\right)+2Cl^{-}\;\left(\mathrm{aq}\right)+2H_{2}O\left(\mathrm{aq}\right)$$

(2) $$\begin{array}{c} \to \mathrm{Mn}\;{\left(\mathrm{OH}\right)}_{2}\;\left(s\right)+2Cl^{-}\;\left(\mathrm{aq}\right)+2H^{+}\left(\mathrm{aq}\right)\\ \left(\mathrm{proton}\;\mathrm{insertion}\;\mathrm{to}\;\mathrm{FeP}O_{4}\mathrm{structure}\right) \end{array}$$

Step 2.

(3) $$\begin{array}{ccc} & & \mathrm{FeP}O_{4}\left(s\right)+\mathrm{xM}n^{2+}\;\left(\mathrm{aq}\right)+yH^{+}\;\left(\mathrm{aq}\right)\;\\ & & \;\to \;H_{y}\mathrm{FeP}O_{4}\left(s\right)+\mathrm{xM}n^{2+}\;\left(\mathrm{aq}\right) \end{array}$$

During this reaction, Mn(OH)₂ is generated during the reduction process. This phenomenon has already been reported in previous literature on Mn‐based batteries.^[^ ^9^, ^11^ ^]^

We conducted XPS measurements during the discharge process at five voltage points (pristine, 0.2, −0.1, −0.5, and −0.9 V vs Ag/AgCl) to further investigate (Figure 3d–f). These XPS profiles offer critical insights into the oxidation states of Fe, Mn, and O. The Fe redox couples and increasing Mn signals are clearly observed in the spectra (Figure 3d,e). The Fe 2p peaks are visible within the 700–745 eV range but disappear during discharge, likely due to the formation of Mn compounds covering the LFP surface. The Mn 2p peaks gradually increase throughout the discharge process, indicating that Mn reacts with the LFP electrode. However, Figure 3b confirms that Mn primarily accumulates on the surface. We also analyzed the O 1s peaks via XPS to verify the formation of Mn(OH)_2_. The pristine sample exhibits a single metal‐oxygen peak at 530.2 eV; however, during discharge, an additional peak emerges at 531.4 eV, which is commonly associated with O─H bonding, suggesting the presence of metal hydrates.

**Figure 4:**
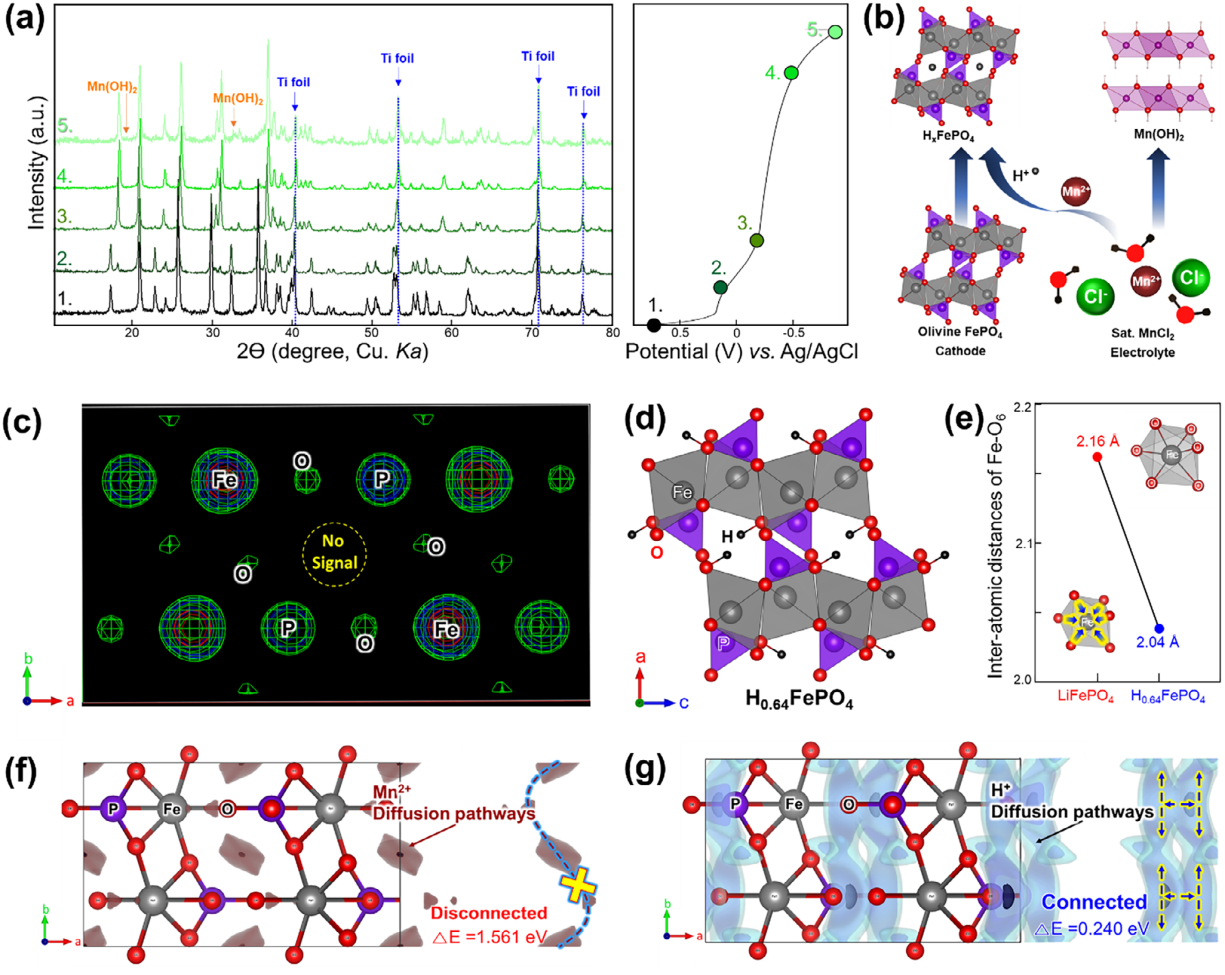
a) XRD profile during the discharge process, along with the corresponding electrochemical profile and b) their reaction mechanism, c) Fourier electron density maps of discharged FePO_4_, d) crystal structure of discharged H_0.64_FePO_4_ e) interatomic distances of Fe─O. f) Mn ion calculated migration paths and barrier, g) proton calculated migration paths and barrier.

We performed XRD measurements during the discharge process to further clarify the charge storage mechanism involving H⁺ insertion (**Figure** **4**a). In the pristine state, only peaks corresponding to LFP and the Ti current collector are observed. However, during the discharge process, new peaks emerge, which can be explained by two primary reaction mechanisms (Figure 4b): 1) The appearance of Mn(OH)_2_ peaks during discharge providing strong evidence for proton insertion into the structure. 2) The disappearance of LFP peaks and the emergence of new ones, indicating that the olivine‐LFP structure undergoes a two‐phase reaction rather than a single‐phase solid solution reaction. The newly generated peaks correspond to the H_0.64_FePO₄ structure. The detailed characterization, including Rietveld refinement results, unit cell parameter changes, proton position, and interatomic distance variations, is presented in Figure 4c–e and the Supporting Information. XRD analysis clearly demonstrates proton insertion into the LFP structure and the formation of Mn(OH)_2_. Additionally, Fourier electron density mapping was applied to the discharged samples. If Mn ions were inserted into the structure, we would expect an increase or the generation of electron density at the 4a site (Figure 4c). However, no signal was detected at the cavity site after discharge, indicating that proton insertion, rather than Mn ion insertion, occurs in the olivine structure. However, this remains an indirect assumption. Therefore, we conducted electrochemical tests (CV and GCD) using a pure HCl aqueous electrolyte (pH 4, matching that of the saturated MnCl₂ aqueous solution). The clearly reversible CV peaks and GCD profiles confirm that charge storage primarily occurs through H⁺ intercalation (Figure S11, Supporting Information). During discharge, the LFP structure undergoes a two‐phase reaction, and the proton‐inserted phase was identified. The phase retains the same orthorhombic *Pnma* structure; however, the unit cell parameters exhibit significant changes compared with the initial structure. The unit cell parameters for the discharged phase (H_0.64_FePO_4_, where 0.64 H is calculated from the discharge capacity) are *a* = 9.815(1) Å, *b* = 5.787(1) Å, and *c* = 4.779(1) Å. The a and b parameters contract by 4.95% and 3.65%, respectively, whereas the c parameter expands by 1.82%. Consequently, the overall unit cell volume decreases from 291.1 Å^3^ to 271.4(1) Å^3^ after discharge.

Additionally, we proposed the crystal structure of H_0.64_FePO_4_ and identified the most stable proton site within the FePO_4_ framework (Figure 4d; Figure S12, Supporting Information). The proton is stably positioned at the 4c site. Table S2 and Figure S13 (Supporting Information) present detailed refinement results for the proton‐inserted phase. When comparing the Fe–O interatomic distances between LFP and H_0.64_FePO_4_, the protonated structure exhibits a distance of 2.04 Å, which is similar to that of FePO_4_. This indicates that proton insertion causes minimal structural distortion during the redox process with a one‐phase reaction. Based on these results, the electrochemical degradation observed during cycling is likely attributed to the formation of inactive Mn(OH)_2_, which acts as a surface blocker, hindering ion transport and negatively impacting performance (Figure 4e).

The diffusion barriers and pathways for Mn^2^⁺ and H⁺ were calculated using the softBV method (Figure 4f,g), with potential migration pathways illustrated along the ab‐plane. Mn^2^⁺ diffusion along the b‐axis was significantly restricted by a high migration barrier of 1.561 eV, indicating that Mn^2^⁺ cannot easily diffuse along this axis (Figure 4f). In contrast, the H⁺ migration barriers, calculated using the same method, revealed several potential pathways along the b‐axis with a much lower migration barrier of 0.240 eV (Figure 4g). The substantially lower migration barriers for H⁺ compared with those of Mn^2^⁺ suggest that proton diffusion is much more favorable within the structure (Figure S14, Supporting Information). This indicates a preference for proton insertion over Mn insertion, leading to side reactions involving Mn, such as the formation of Mn(OH)_2_.

We have uncovered the previously unknown reaction mechanism of LFP in MnCl₂ electrolyte solution. XRD analysis clearly revealed structural changes, providing strong evidence that the LFP structure is actively involved in the charge storage reaction. During charge–discharge cycling, Mn(OH)₂ peaks were observed in the XRD patterns, a phenomenon known to occur when protons intercalate into the LFP structure. To verify whether manganese or proton was inserted into the discharged phase, we conducted Fourier electron density map analysis. The absence of a manganese signal within the structure confirms that the primary charge carrier is proton (H⁺). Additionally, diffusion barrier calculations further support that protons diffuse and intercalate more favorably into the structure compared to Mn^2^⁺. Based on these findings, we propose the most stable site for proton storage within the LFP framework.

### 2.4 Demonstration of Hybrid Li^+^/Mn^2+^ Aqueous Battery System

**Figure 5:**
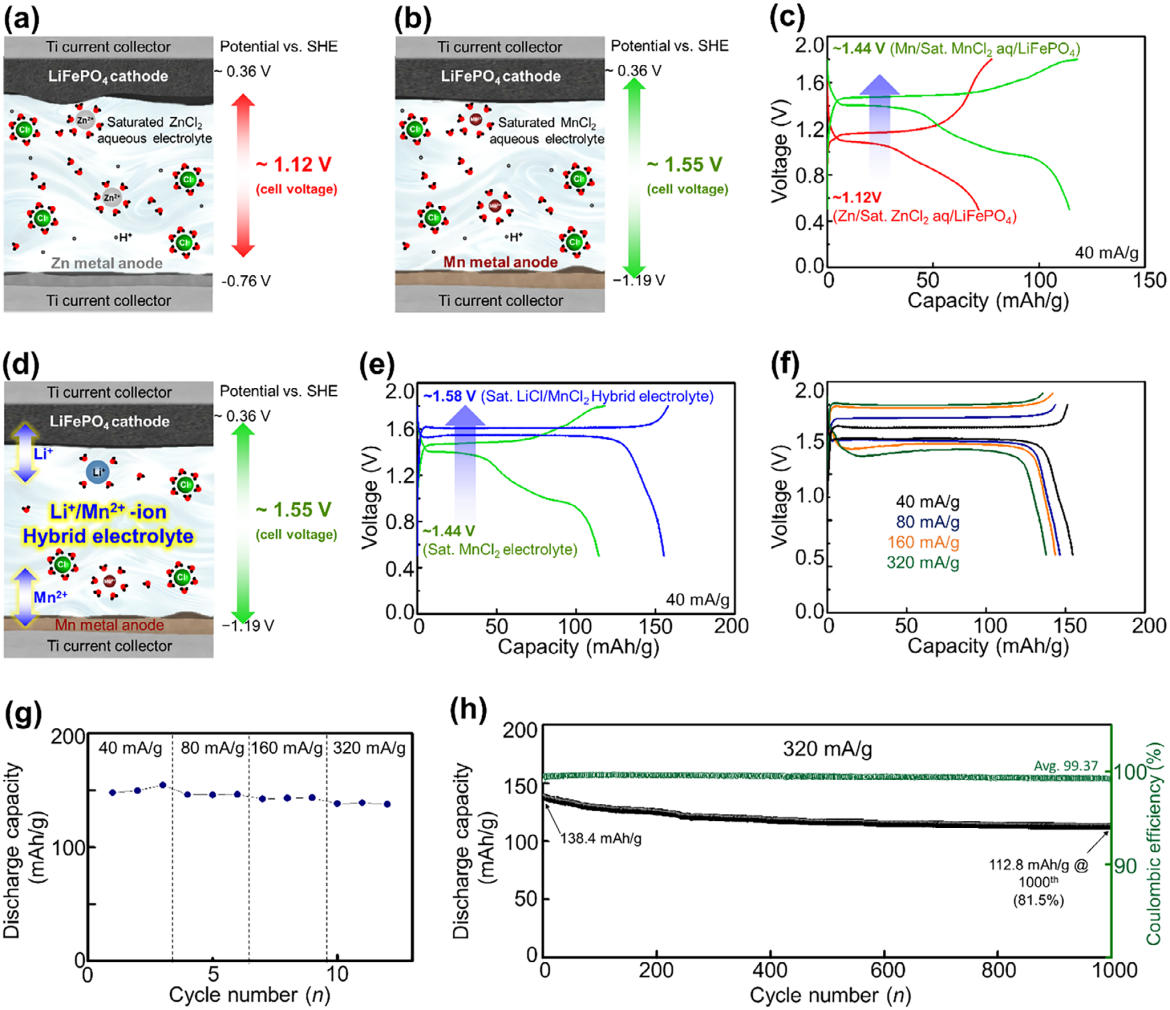
Schematic illustration of a) [Zn/sat. ZnCl_2_ aq./LFP], and b) [Mn/sat. MnCl_2_ aq./LFP] cell, c) initial charge–discharge curves at 40 mA g^−1^ for Zn and Mn cell with LFP cathode, d) Schematic illustration of hybrid type cell using Mn and Li^+^/Mn^2+^ electrolyte with LFP cathode, electrochemical characterizations of [Mn/sat. 1LiCl/1MnCl_2_ aq./LFP] cell e) initial charge–discharge curves at 40 mA g^−1^, f) GDC profile under various current densities, g) Rate performance at different current densities, h) Long term cyclability at 320 mA g^−1^.

A comparison was made with the well‐established Zn battery system to explore the potential of the proposed Mn‐based battery system as a next‐generation technology. Owing to the relatively high redox potential of Zn (−0.76 V vs SHE), the output voltage of Zn‐based batteries is lower. Specifically, when combined with an LFP cathode, the theoretical output voltage is ≈1.12 V (**Figure** **5**a). However, when the Zn counter electrode is replaced with the Mn counter electrode proposed in this study, Mn, with a lower redox potential (−1.19 V vs SHE), increases the operating voltage by ≈0.43 V, resulting in an operating voltage of 1.55 V. We conducted tests in two‐electrode systems using ZnCl_2_ and MnCl_2_ electrolytes to validate this finding (Figure 5b). The Zn‐based system exhibited an operating voltage of ≈1.12 V, whereas the Mn‐based system exhibited a higher voltage of 1.44 V, confirming its potential as a next‐generation aqueous secondary battery system (Figure 5c). However, when constructing an Mn‐based battery using LFP as the cathode, the reaction mechanism involves proton insertion in the previous section, which promotes electrolyte acidity and side reactions that degrade battery performance.

In addition, the electrochemical performance of LFP/Mn hybrid batteries using only Mn‐based electrolytes was unsatisfactory. This challenge prompted us to explore a new system, ultimately leading to a breakthrough. We propose a hybrid battery utilizing Mn metal counter electrode for the first time to overcome this limitation. When Li and Mn salts are used, the system maintains its inherent voltage and improves the electrochemical performance and cycling stability of the cathode (Figure 5d). Figure 5e compares the performance of the pure Mn/LFP system [Mn/sat. MnCl_2_ aq./LFP] with the hybrid Mn/LFP system [Mn/sat. 1LiCl/1MnCl_2_ aq./LFP], demonstrating enhanced overall electrochemical performance with the hybrid configuration. The operating voltage exhibits 1.58 V with ≈155 mA g^−1^ of capacity. The hybrid system demonstrated excellent rate capability, with reversible discharge capacities of 155, 146, 142, and 138 mAh g^−1^ at current densities of 40, 80, 160, and 320 mA g^−1^, respectively (Figure 5f,g). At a current density of 320 mA g^−1^, the discharge capacity after long‐term cycling began at 138.4 mAh g^−1^, retaining 81.5% of its capacity (112.8 mAh g^−1^) after 1000 cycles, demonstrating extremely durable cycles with enhanced overall electrochemical performances (Figure 5h). Over 1000 cycles, the average Coulombic efficiency remained high at 99.37%, indicating minimal electrolyte decomposition. The detailed CV and GDC curves are presented in Figures S15 and S16 (Supporting Information).

To develop a practical hybrid Li⁺/Mn^2^⁺ aqueous battery system, further research on electrolytes and the discovery of new cathode materials are essential. This study is the first to demonstrate the successful operation of a hybrid Li⁺/Mn^2^⁺ aqueous battery system. Moreover, achieving an output of 1.6 V with a capacity of ≈150 mAh g^−1^ in an aqueous system represents a significant advancement in the field.

Further research is needed to fully understand the working mechanism of LFP in the Li⁺/Mn^2^⁺ hybrid system. This study serves as a starting point, and we plan to conduct additional investigations in the future.

## 3 Conclusion

In this study, we explored LFP as a cathode material for aqueous Mn‐based hybrid batteries and investigated the H⁺ storage mechanism through electrochemical characterization, advanced structural spectroscopy, and diffusion pathway analysis. The material exhibited a reversible capacity of ≈109.2 mAh g^−1^ at 40 mA g^−1^ with the cycle retention of 42.1% after 3000 cycles at 320 mA g^−1^ in a saturated MnCl_2_ aqueous electrolyte. Protons, which possess a lower migration barrier than Mn ions, were more easily and reversibly inserted into the LFP structure, leading to Mn(OH)_2_ formation. The Fourier electron density map clearly indicated that Mn ions did not intercalate into the LFP structure. However, the cycling performance remained inadequate, prompting us to introduce a novel Mn^2^⁺/Li⁺ hybrid battery. This system achieved an operating voltage of 1.58 V and an improved reversible capacity of ≈155.3 mAh g^−1^ at 40 mA g^−1^, along with considerably enhanced cycle stability, retaining 81.5% capacity after 1000 cycles.

Here, we propose a promising next‐generation aqueous battery system. Mn‐based hybrid batteries offer higher theoretical energy density than Zn‐based systems, but advanced electrolyte engineering is needed to suppress hydrogen evolution at the Mn counter electrode. A hybrid system can further enhance energy density, emphasizing the need for future research on electrolyte optimization. Especially, to achieve high‐energy‐density systems, further exploration of new cathode materials remains essential. These findings underscore the potential of Mn‐based batteries for aqueous energy storage applications.

## 4 Experimental Section

### Material Synthesis and Characterization of LFP

LFP powders were synthesized using a sol–gel method with LiOH, Fe(CH_3_COO)_2_, and NH_3_H_2_PO_4_ (all from Sigma Aldrich). In a typical procedure, the chemicals were mixed in water at the appropriate molar ratios and then dried at 60 °C to remove the solvent. The resulting mixture was initially heated at 300 °C under an Ar flow, followed by calcination at 650 °C for 6 h under an Ar atmosphere. Morphological characterization was conducted using SEM (Hitachi 8020) and TEM (FEI, Themis Z) equipped with EDX for elemental analysis. Structural analysis was performed using a Rigaku Mini‐Flex 600 X‐ray diffractometer with Cu Kα radiation (λ = 1.542 Å). Rietveld refinement was performed using the GSAS software^[^ ^18^ ^]^ to confirm the LFP phase.

### Electrochemical Test

The LFP cathodes were prepared by mixing LFP powder, Super P carbon (Timcal), and PVDF binder (Kureha Co.) in a weight ratio of 8:1:1. This mixture was dispersed in N‐methyl‐2‐pyrrolidone solution and coated onto 32 µm Ti foil (Sigma–Aldrich). The coated electrodes were dried at 80 °C and pressed using an electrode press. For the three‐electrode cell test, activated carbon was employed as the counter electrode to prevent hydrogen evolution reaction (HER) at the Mn counter electrode, while Ag/AgCl was used as the reference electrode. A glass fiber separator (Whatman) was used to separate the electrodes. A saturated MnCl_2_ solution (Sigma–Aldrich) in deionized water served as the aqueous electrolyte. For the two‐electrode system, Zn and Mn foils were utilized as the counter electrode. Electrochemical characterizations were performed using a Biologic VMP‐3e, and impedance measurements were conducted in PEIS mode across a frequency range of 0.1–200 kHz.

### Structural Analyses

The discharged H₀.₆₄FePO₄ structure was analyzed using advanced structural determination techniques. First, Le Bail fitting was applied using GSAS software^[^ ^18^ ^]^ to refine the diffraction pattern. Then, structure factors (including peak positions, structural phase, and intensity information) were extracted and further refined using the single‐crystal analysis program Crystals.^[^ ^19^ ^]^ Finally, Fourier transform was applied, and the structure was visualized using the MCE program.^[^ ^20^ ^]^

### Ion Migration‐Barrier Calculation

The Mn^2^⁺ and H⁺ migration barriers were calculated using the softBV program.^[^ ^21^ ^]^ Migration characteristics for Mn^2^⁺ and H⁺ were analyzed based on the crystal structure derived from the LFP structure, as obtained through Rietveld refinement of the powder XRD data using the GSAS software. The 3D migration pathways were visualized using VESTA (version 3).^[^ ^22^ ^]^

## Conflict of Interest

The authors declare no conflict of interest.

## Supporting information



Supporting Information

## Data Availability

The data that support the findings of this study are available from the corresponding author upon reasonable request.
