# Supplementary material for: The Charge Storage Mechanism and Durable Operation in Olivine–Lithium–Iron–Phosphate for Mn‐based Hybrid Batteries
Source: Adv Sci (Weinh). 2025 Mar 17;12(19):2502866. doi: 10.1002/advs.202502866 (PMC12097060; doi:10.1002/advs.202502866)
Supplement: Supplementary file 1 — Supporting Information [file ADVS-12-2502866-s001.docx]

Supporting Information

**The Charge Storage Mechanism and Durable Operation in Olivine-Lithium-Iron-Phosphate for Mn-based Hybrid Batteries**

*Jangwook Pyun, Hyungjin Lee, Hyeonjun Lee, Sangki Lee, Seunghyeop Baek, Hyeju Kwon, Seung-Tae Hong, Munseok S. Chae**

**
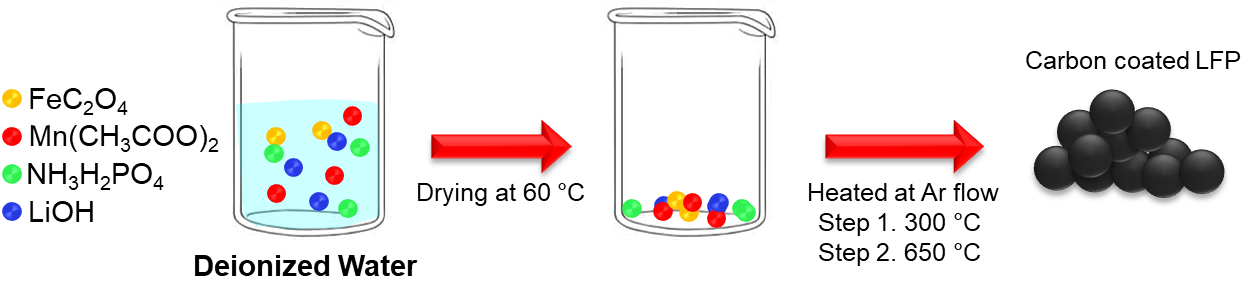
**

**Figure S1.** Schematic diagram of the carbon-coated LFP synthesis process.

**Table S1.** Powder XRD Rietveld refinement results for LiFePO_4_: atomic coordinates, site occupancies, and reliability factors at room temperature.

| Crystal System  Space Group  Lattice Parameters, Volume, Z | | | | Orthorhombic  *P n m a (No. 62)*  *a* = [10.326(1)](P_publ%20_cell_length_a) Å  *b* = [6.006(1)](P_publ%20_cell_length_b) Å  *c* = [4.693(1)](P_publ%20_cell_length_c) Å  α, β, γ = 90 ^o^  V = 291.1(1) Å^3^, *Z* = 4 | | | |
| --- | --- | --- | --- | --- | --- | --- | --- |
| Atoms | *x* | *y* | *z* | | Wyckoff | Occupancy | U_iso_×100 |
| Li(1) | 0.0000 | 0.0000 | 0.0000 | | 4*a* | 1.00 | 1.0(1) |
| Fe(1) | 0.2182(1) | 0.2500 | 0.5270(1) | | 4*c* | 1.00 | 1.0(1) |
| P(1) | 0.0932(1) | 0.7500 | 0.5785(1) | | 4*c* | 1.00 | 1.0(1) |
| O(1) | 0.1652(1) | 0.5449(1) | 0.7109(1) | | *8d* | 1.00 | 1.0(1) |
| O(2) | 0.0436(1) | 0.2500 | 0.2873(1) | | 4*c* | 1.00 | 1.0(1) |
| O(3) | 0.0935(1) | 0.7500 | 0.2524(1) | | 4*c* | 1.00 | 1.0(1) |

*R*_p_ = [0.195](P_publ%20_pd_proc_ls_prof_R_factor), *R*_wp_ = [0.261](P_publ%20_pd_proc_ls_prof_wR_factor), *R*_exp_ = [0.235](P_publ%20_pd_proc_ls_prof_wR_expected), *R*(*F*^2^) = [0.221](P_publ%20_refine_ls_R_Fsqd_factor), χ^2^ = 1.23

**
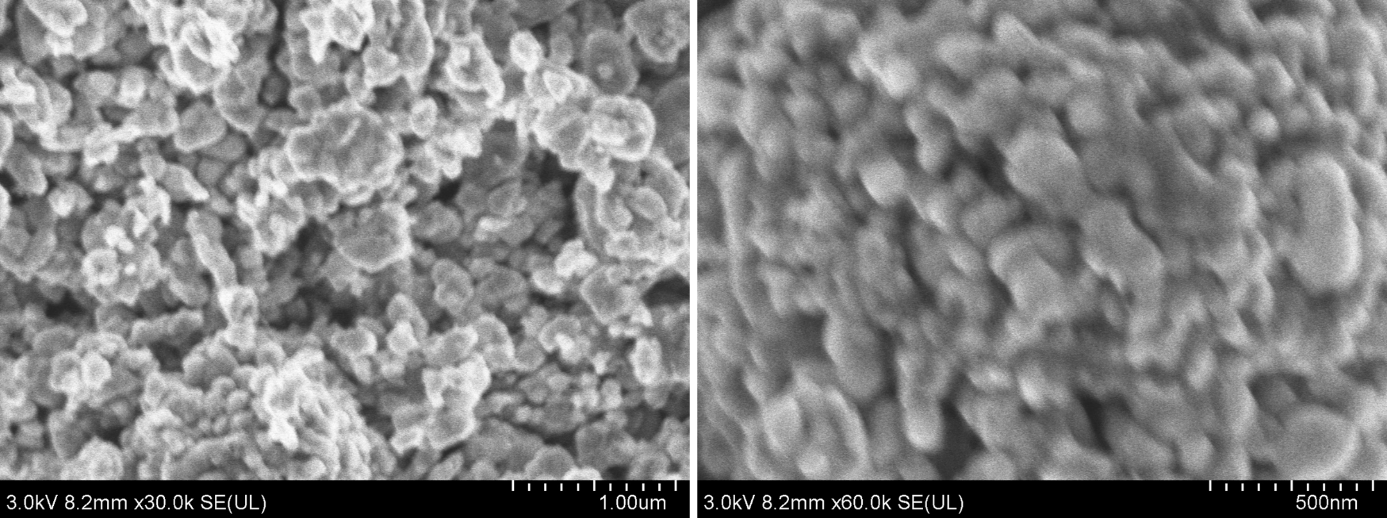
**

**Figure S2.** SEM images of as-prepared LiFePO_4._


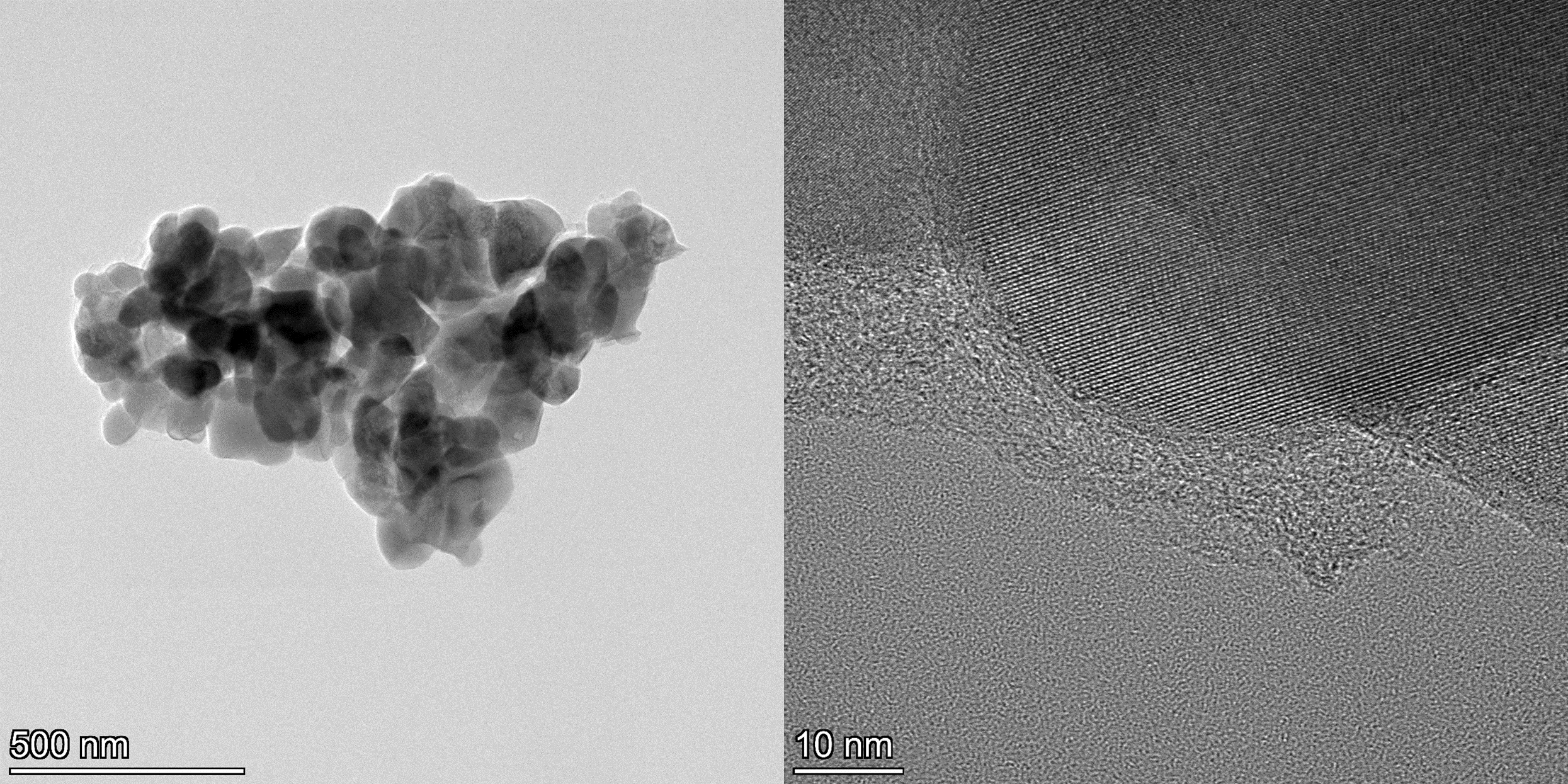


**Figure S3.** TEM images of as-prepared LiFePO_4._

**
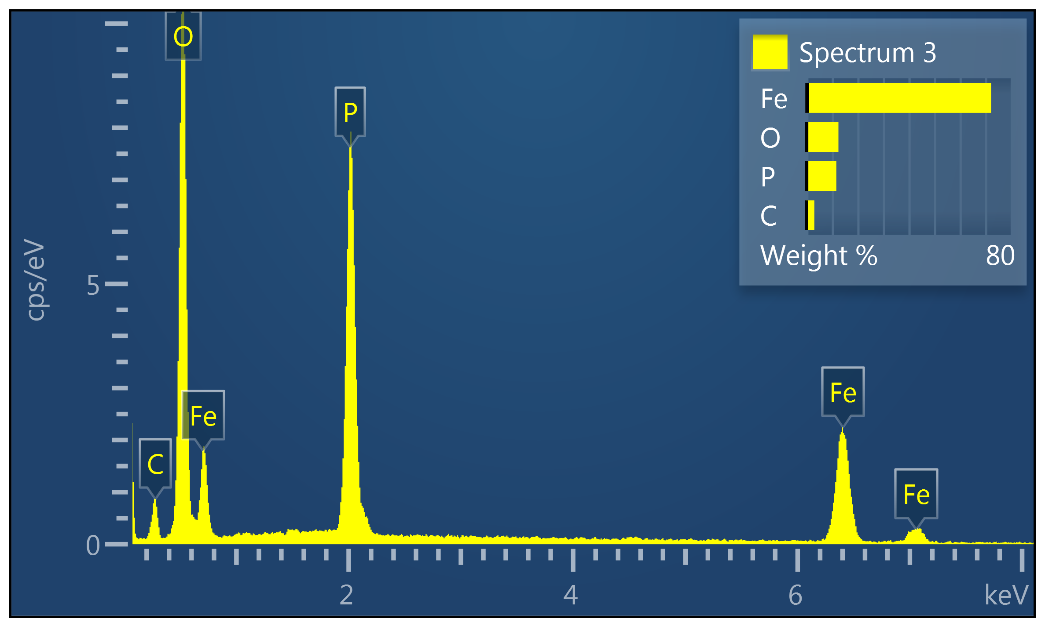
**

**Figure S4.** SEM-EDX spectra of as-prepared LiFePO_4._


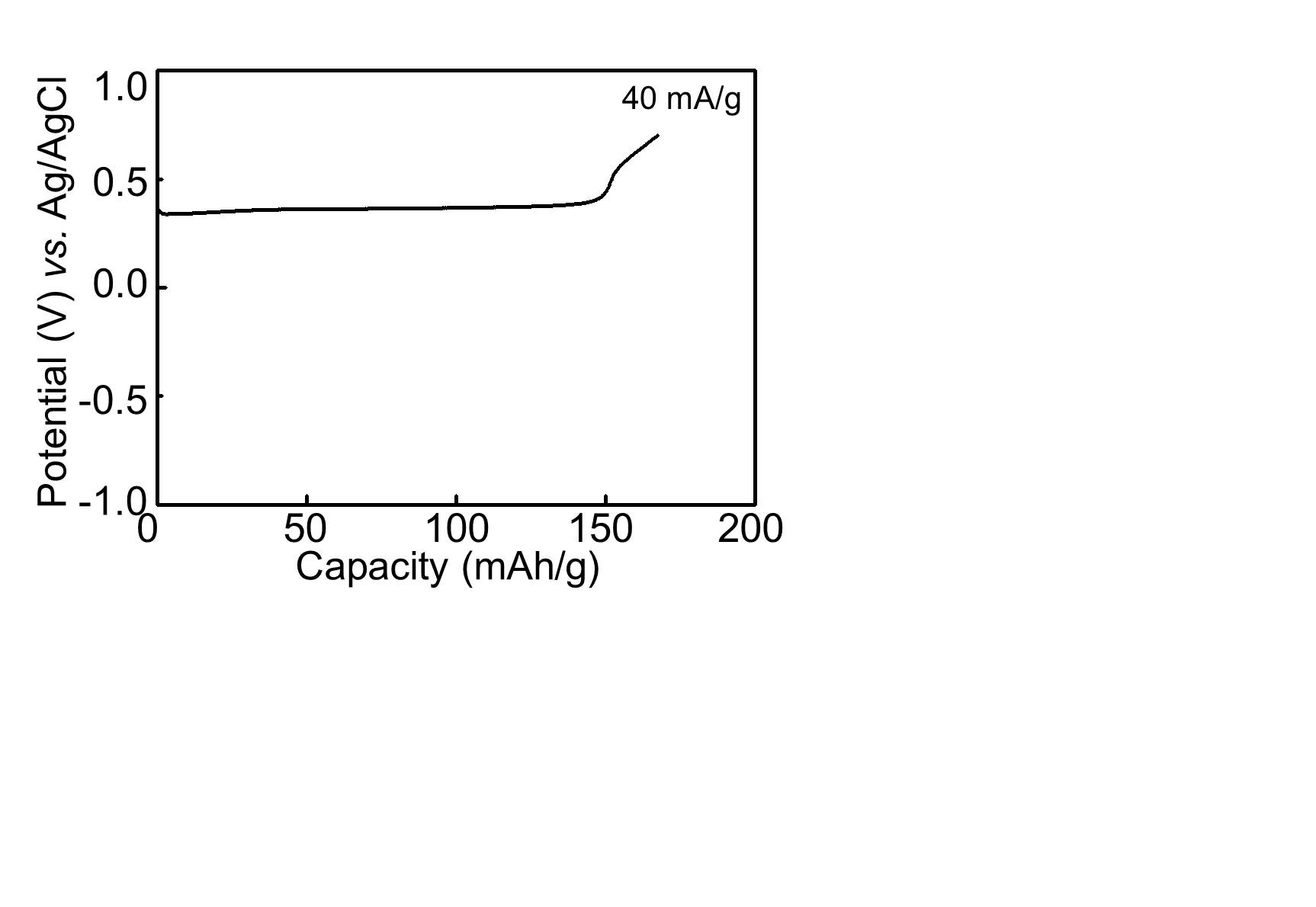


**Figure S5.** The initial charge profile of LiFePO_4_ under 40 mA/g current.

**
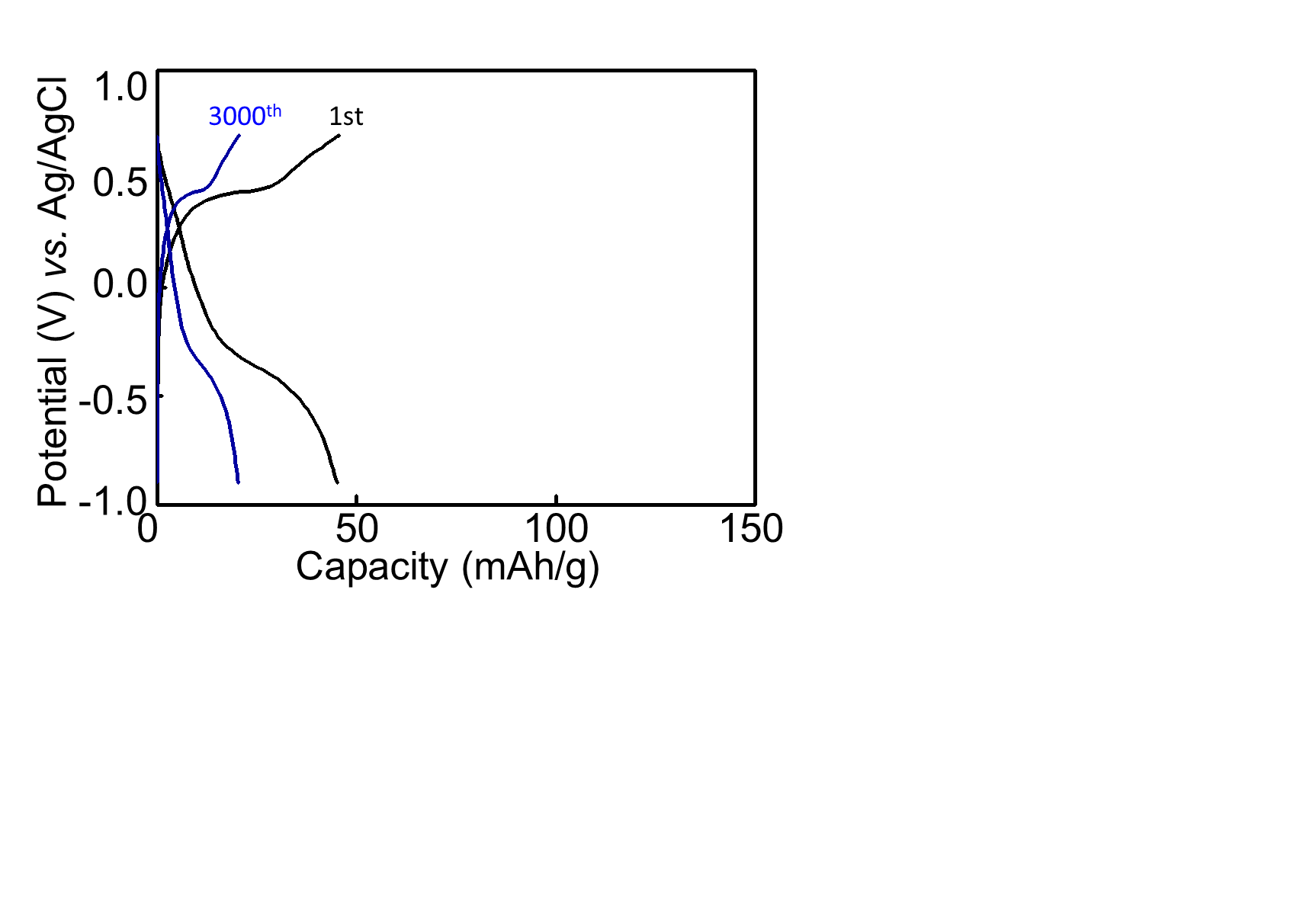
**

**Figure S6.** Galvanostatic charge/discharge curves for initial and 3000^th^ cycles under 320 mA/g current.


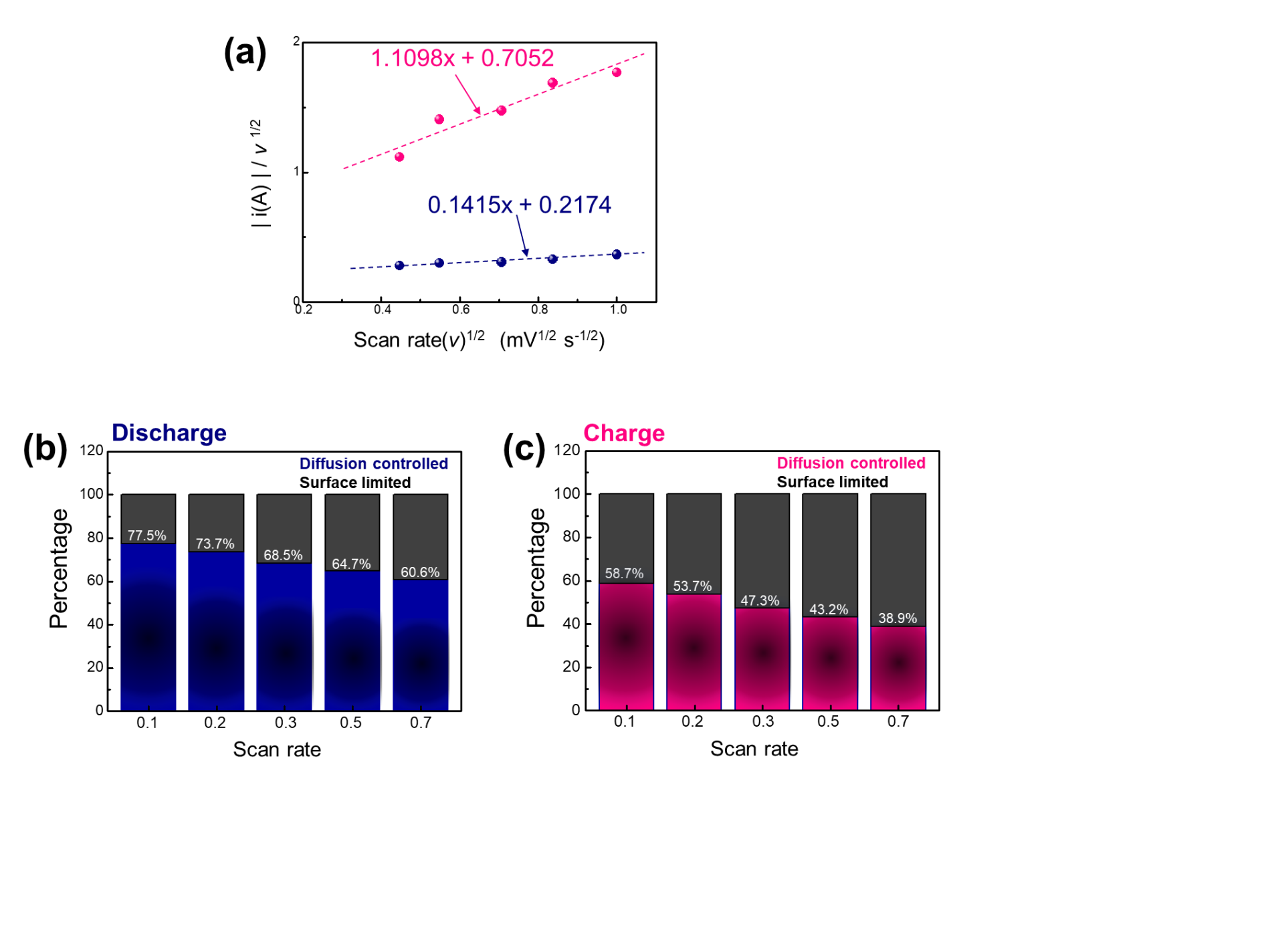


**Figure S7.** (a) Cathodic peak current dependence on the scan rate obtained to determine the capacitive and intercalation contributions to energy storage. Calculated intercalation/adsorption ratios with various scan rates for (b) discharge and (c) charge process.


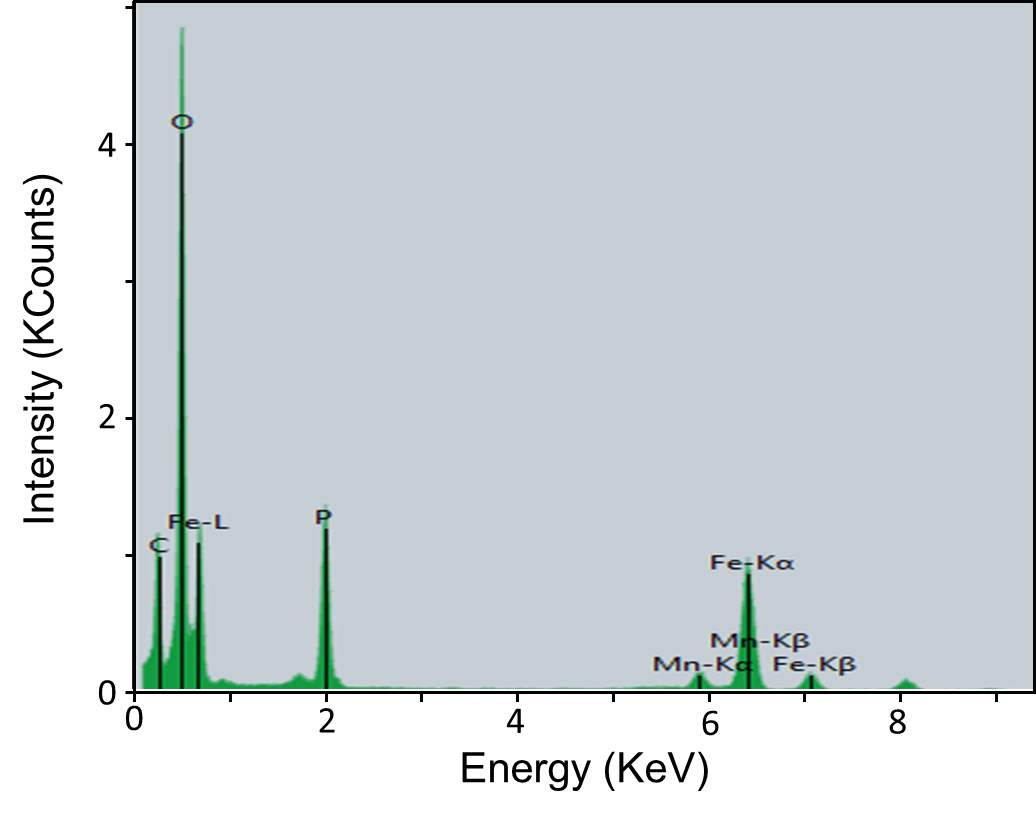


**Figure S8.** TEM-EDX spectra of the discharged electrode.


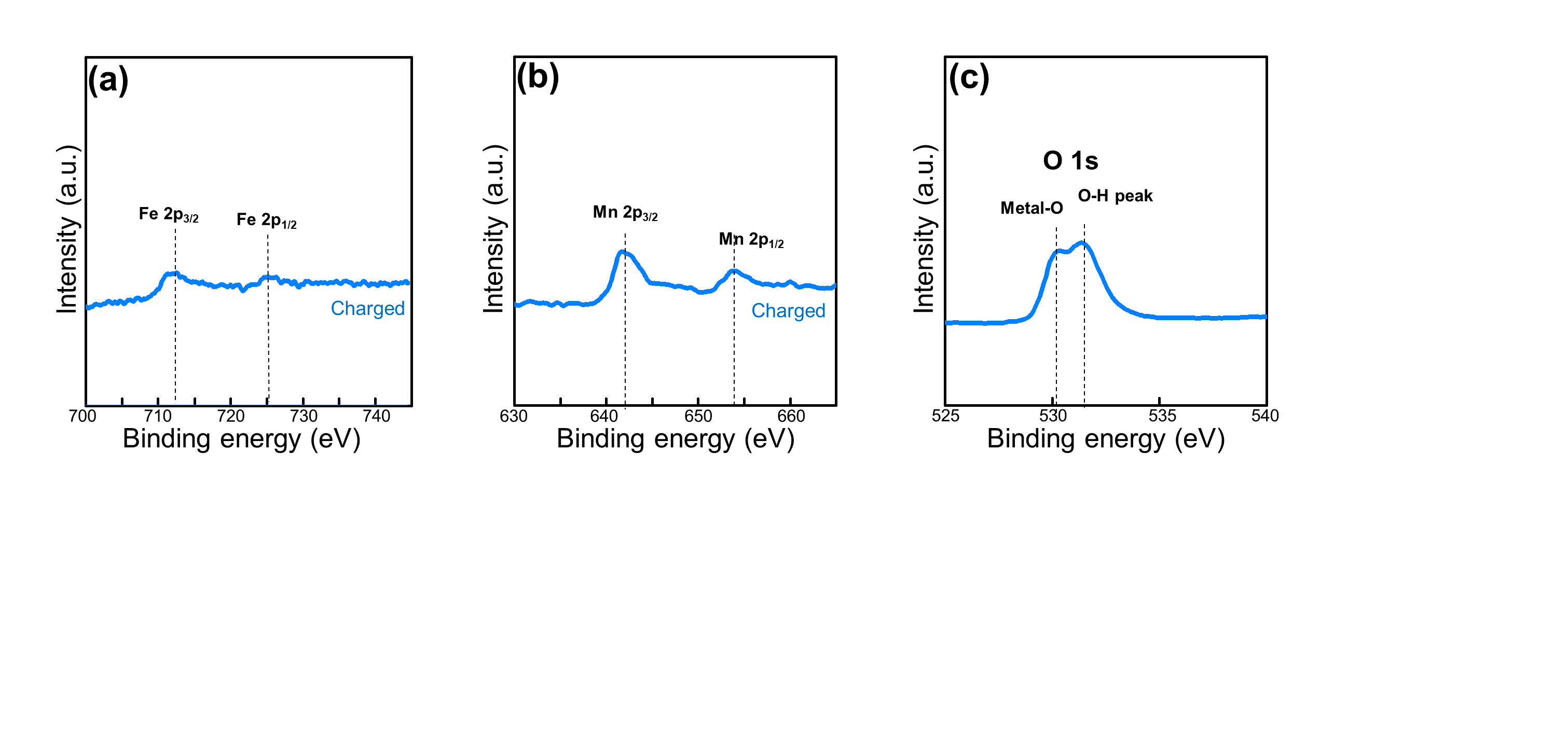


**Figure S9.** XPS spectra of the charged LFP electrode in a saturated MnCl₂ aqueous electrolyte solution.


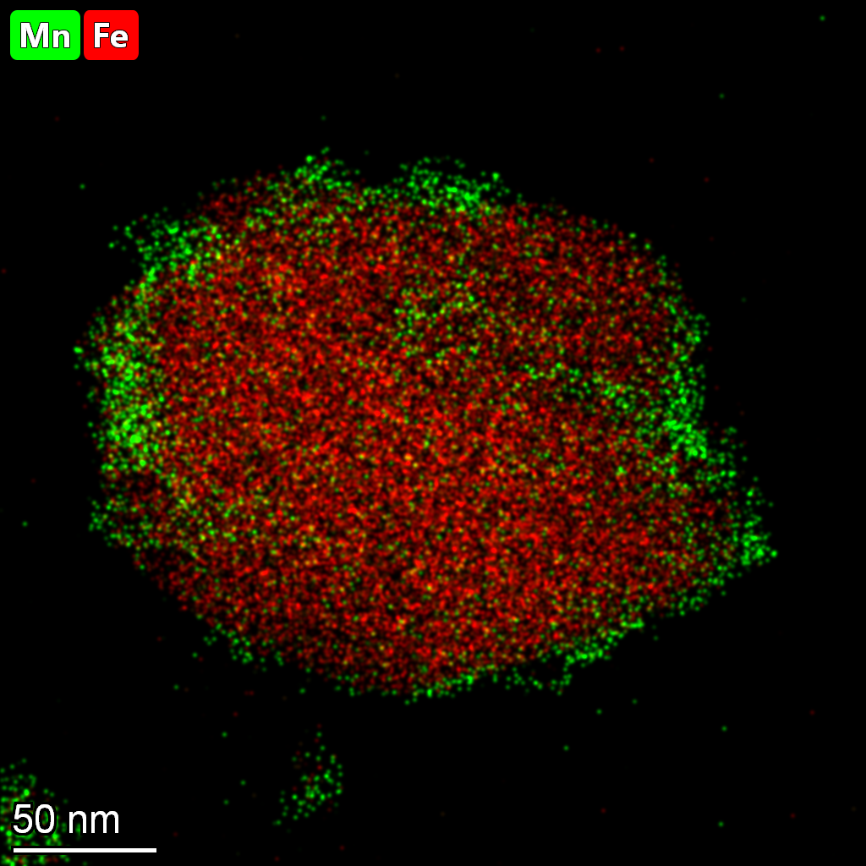


**Figure S10.** TEM-EDX elemental mapping of the discharged sample.


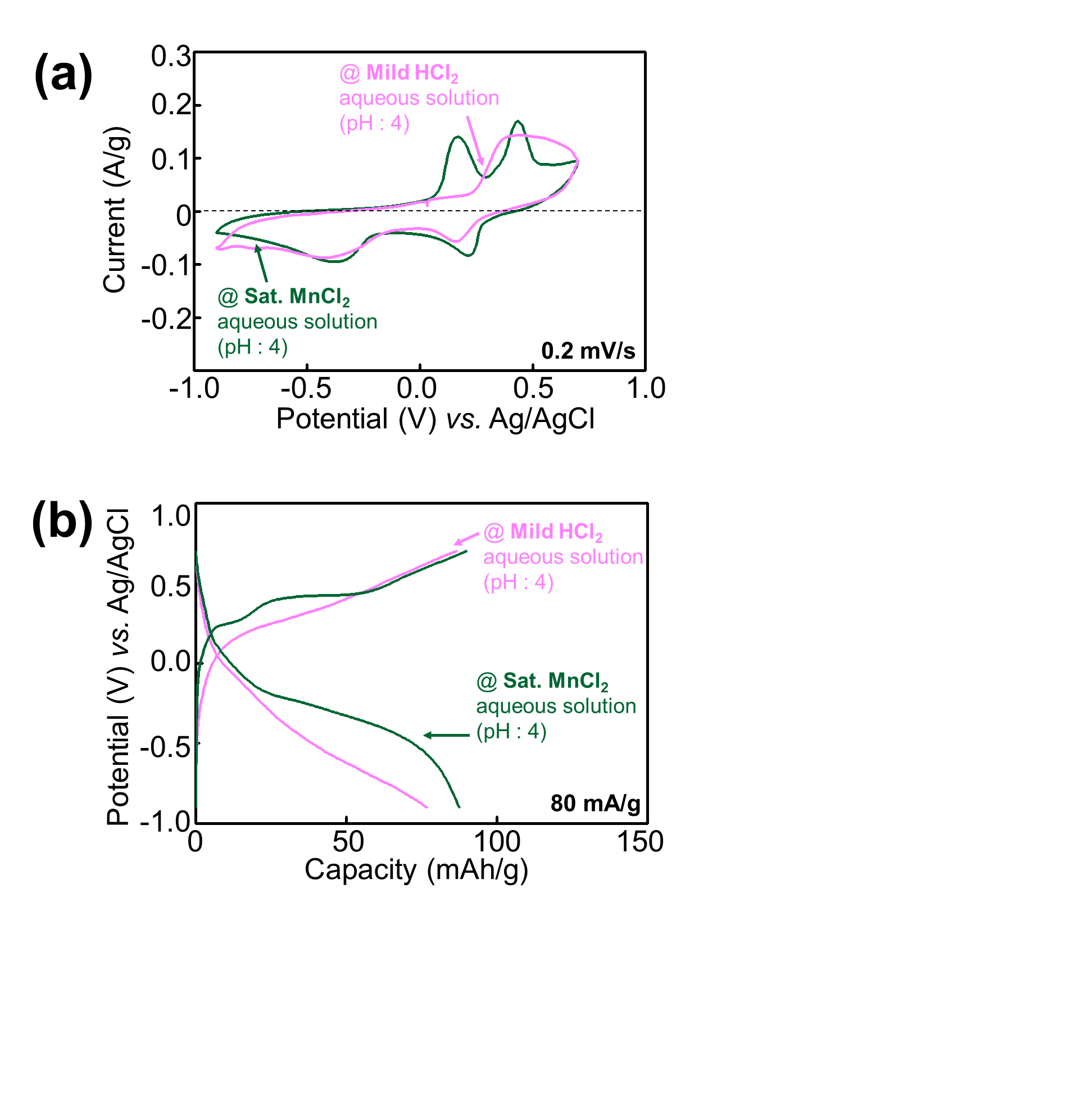


**Figure S11.** Electrochemical reduction and oxidation profiles of the LFP electrode in a mild HCl aqueous solution (pH 4) and a saturated MnCl₂ aqueous electrolyte (pH 4): (a) CV curves and (b) GCD profiles.


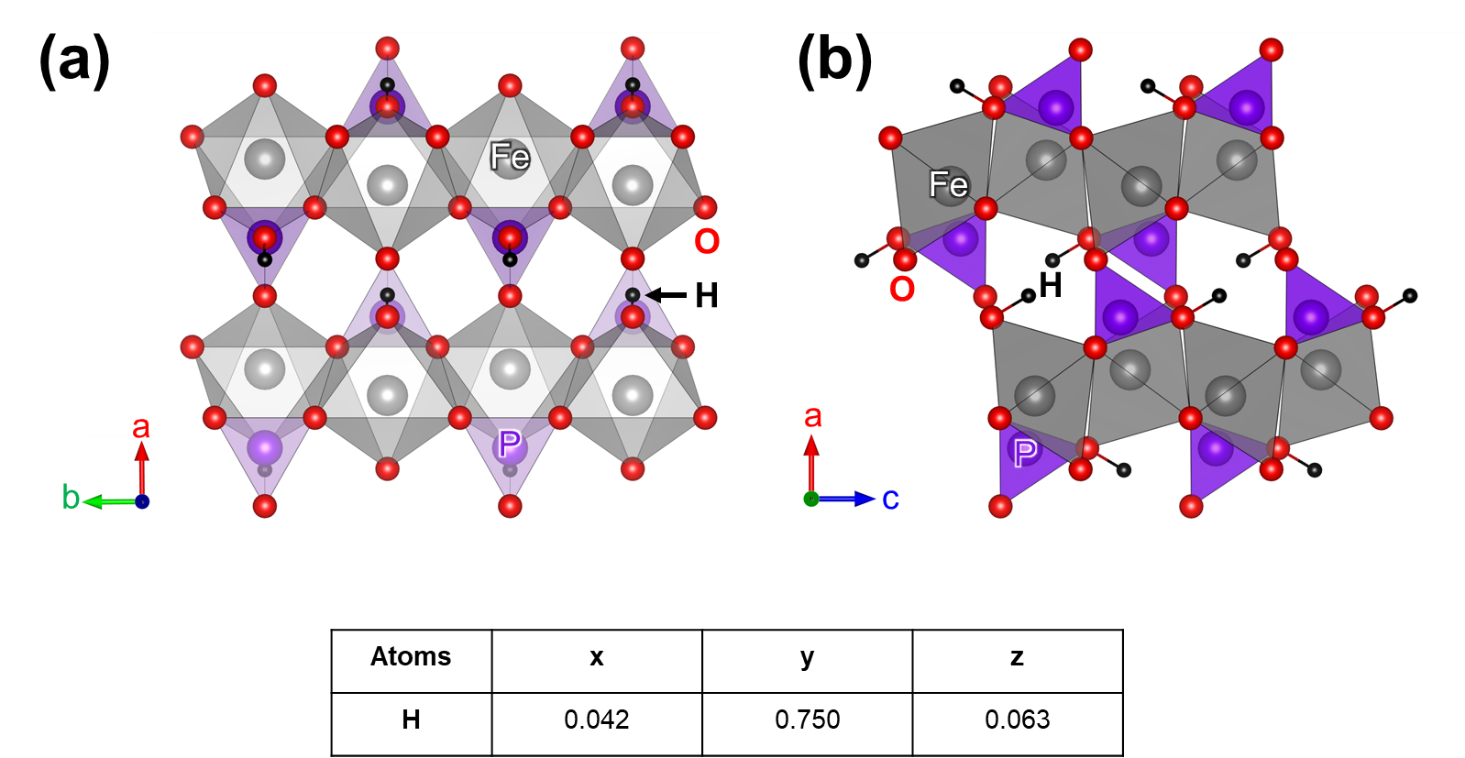


**Figure S12.** Calculation results for stable H^+^ site after discharge: atomic coordinates and structure images (a) ab-plane, (b) ac-plane.

**Table S2.** Powder XRD Rietveld refinement results for H_0.64_FePO_4_: atomic coordinates, site occupancies, and reliability factors at room temperature.

| Crystal System  Space Group  Lattice Parameters, Volume, Z | | | | Orthorhombic  *P n m a (No. 62)*  *a* = 9.8146(8)Å  *b* = 5.7870(4)Å  *c* = 4.7786(5)Å  α, β, γ = 90 ^o^  V = 271.4(1) Å^3^, *Z* = 4 | | | |
| --- | --- | --- | --- | --- | --- | --- | --- |
| Atoms | *x* | *y* | *z* | | Wyckoff | Occupancy | U_iso_×100 |
| H(1) | 0.0400(2) | 0.75 | 0.0610(2) | | 4*c* | 0.64 | 3.3(1) |
| Fe(1) | 0.2240(2) | 0.2500 | 0.5472(2) | | 4*c* | 1.00 | 3.3(1) |
| P(1) | 0.0918(2) | 0.7500 | 0.5967(2) | | 4*c* | 1.00 | 3.3(1) |
| O(1) | 0.1651(2) | 0.54423(24) | 0.7452(2) | | *8d* | 1.00 | 3.3(1) |
| O(2) | 0.0553(2) | 0.2500 | 0.3449(2) | | 4*c* | 1.00 | 3.3(1) |
| O(3) | 0.1184(2) | 0.7500 | 0.2547(2) | | 4*c* | 1.00 | 3.3(1) |

* R_p_ = [0.142](D_p_01%20_pd_proc_ls_prof_R_factor), R_wp_ = [0.194](D_p_01%20_pd_proc_ls_prof_wR_factor), R_exp_ = [0.199](D_p_01%20_pd_proc_ls_prof_wR_expected), R(F^2^) = [0.118](D_p_01%20_refine_ls_R_Fsqd_factor), χ^2^ = 0.960


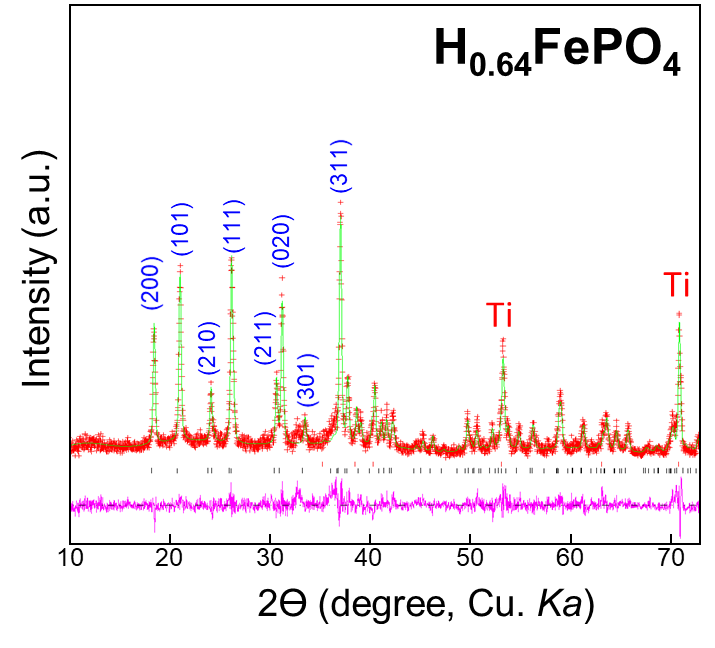


**Figure S13.** Rietveld results discharged electrode H_0.64_FePO_4._ *0.64 H are calculated from the discharge capacity

**
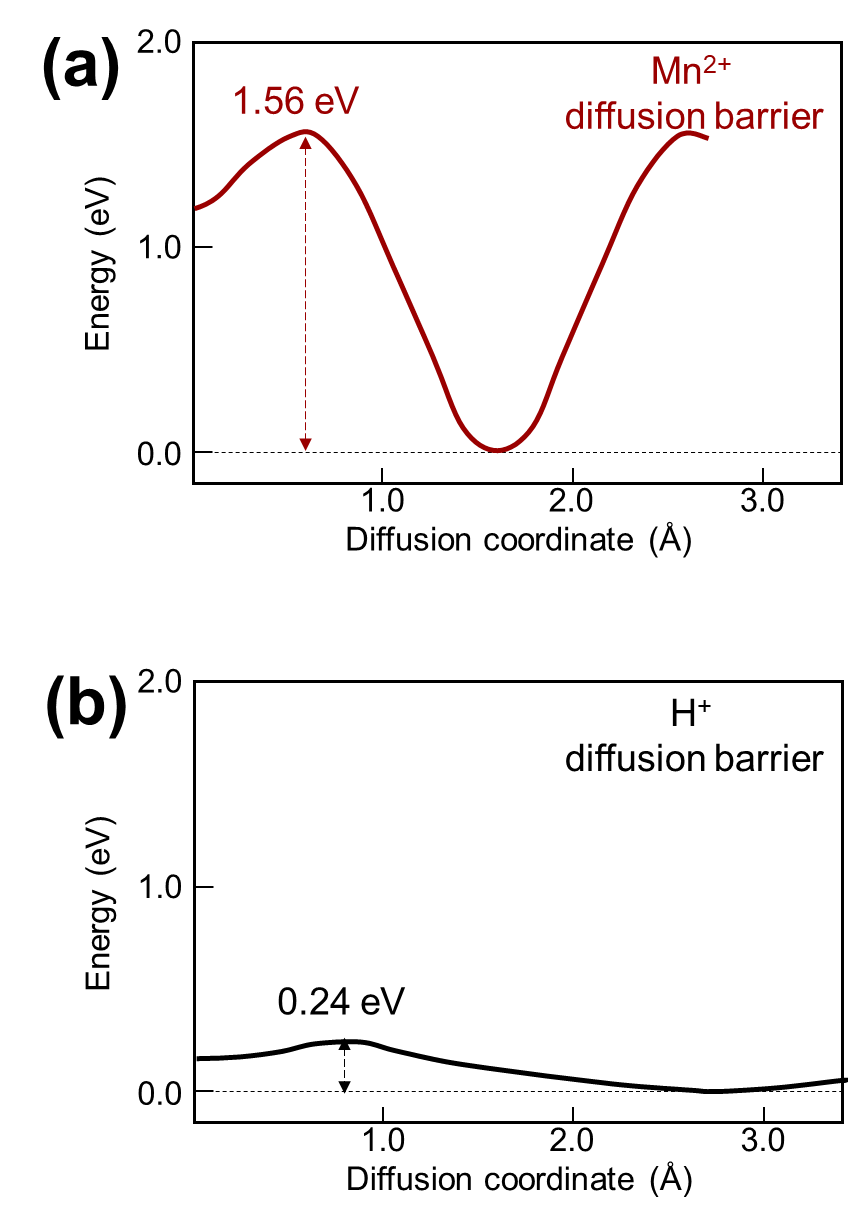
**

**Figure S14.** Migration barrier of (a) Mn^2+^ and (b) H^+^ in the FePO_4_ structure.

**
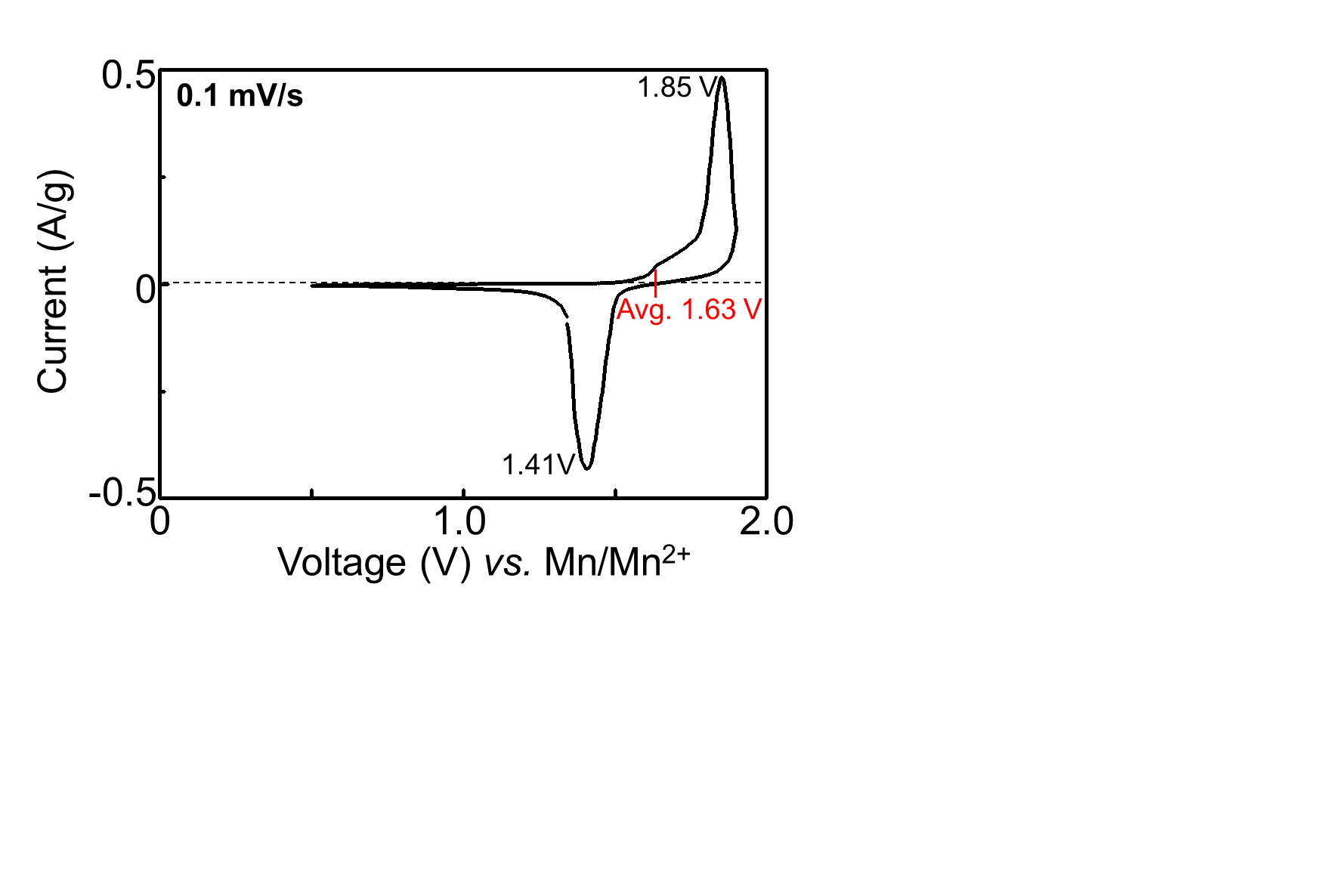
**

**Figure S15.** CV curve of [Mn/sat. 1LiCl/1MnCl_2_ aq./LFP] hybrid cell in scan rate 0.1 mV/s.


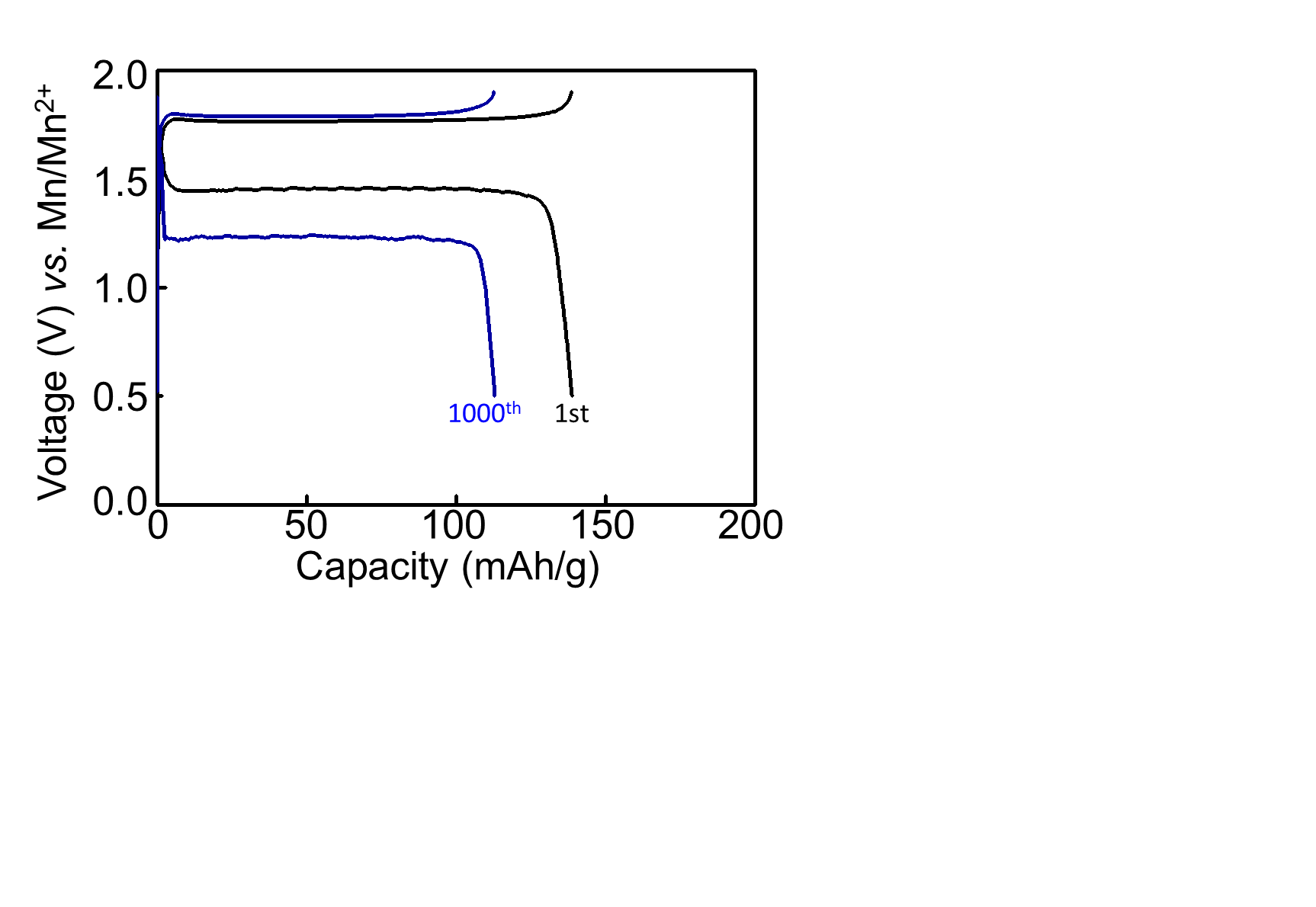


**Figure S16.** Galvanostatic charge-discharge curves of the [Mn/sat. 1LiCl/1MnCl₂ aq./LFP] hybrid cell, recorded for both the initial cycle and the 3000th cycle at a current density of 320 mA/g.
